# Neuropsychiatric manifestations of SARS-CoV-2 infection

**DOI:** 10.1192/j.eurpsy.2021.297

**Published:** 2021-08-13

**Authors:** V. Nogueira, M. Marguilho, I. Pereira, J. Teixeira, M. Mendes

**Affiliations:** 1 Clínica 4 - Unidade De Alcoologia E Novas Dependências, Centro Hospitalar Psiquiátrico de Lisboa, Lisboa, Portugal; 2 Clínica 5, Centro Hospitalar Psiquiátrico de Lisboa, Lisboa, Portugal

**Keywords:** SARS-CoV-2, nervous system, COVID-19

## Abstract

**Introduction:**

Starting in December 2019, the coronavirus SARS-CoV-2 emerged and soon acquired a pandemic dimension. The evidence that 1 in 3 patients presented neuropsychiatric symptoms highlighted SARS-CoV-2 neurotropic properties. The involvement of the Central Nervous System (CNS) seems to be associated with poor prognosis, and it can occur independently of the respiratory system.

**Objectives:**

To assess neuropsychiatric symptoms in SARS-CoV-2 patients and possible mechanisms of CNS invasion; to reflect on what changes should be made in order to avoid short and long-term complications.

**Methods:**

A non-systematic literature review was performed, including publications between January and August 2020.

**Results:**

The most frequent CNS presentations included fatigue (38-75%), headache (6,5-34%), nausea or vomiting (1-13,7%). Regarding PNS involvement, three kinds of hypoesthesia (hyposmia, hypogeusia, and hypopsia) were commonly present. Additionally, cases of neurological syndromes associated with SARS-CoV2 were reported, being related to a poor prognosis in cases such as brainstem infiltration. Another major concern regarding CNS involvement is the possibility of permanent neurological disabilities. Importantly there are reports of patients who tested positive for SARS-CoV-2 in CFS, without samples from nasopharyngeal swabs. Different hypothesis are postulated to explain possible mechanisms through which SARS-CoV-2 affects CNS, including: direct invasion through the olfactory nerve, hematogenous route through ACE-2 (angiotensin-converting enzyme) receptor expressed in blood-brain-barrier; or indirect mechanisms.

**Conclusions:**

Here we discuss the neuropsychiatric manifestations of SARS-CoV-2 infection and the potential mechanisms by which they occur at an early stage. Awareness, prevention and early treatment of potential neuropsychiatric symptoms of COVID-19 should not be overlooked, especially because they seem to predict a worse prognosis.

**Disclosure:**

No significant relationships.

